# Slack, Slick, and Sodium-Activated Potassium Channels

**DOI:** 10.1155/2013/354262

**Published:** 2013-05-13

**Authors:** Leonard K. Kaczmarek

**Affiliations:** Departments of Pharmacology and Cellular and Molecular Physiology, Yale University School of Medicine, 333 Cedar Street, New Haven, CT 06520-8066, USA

## Abstract

The *Slack* and *Slick* genes encode potassium channels that are very widely expressed in the central nervous system. These channels are activated by elevations in intracellular sodium, such as those that occur during trains of one or more action potentials, or following activation of nonselective cationic neurotransmitter receptors such as AMPA receptors. This review covers the cellular and molecular properties of Slack and Slick channels and compares them with findings on the properties of sodium-activated potassium currents (termed K_Na_ currents) in native neurons. Human mutations in Slack channels produce extremely severe defects in learning and development, suggesting that K_Na_ channels play a central role in neuronal plasticity and intellectual function.

## 1. Introduction

 A key determinant of the many varied types of firing patterns that can be observed in excitable cells is the number and types of potassium channels that are expressed in the plasma membrane. In contrast to the relatively low number of genes for other channels that regulate excitability (10 known genes each for voltage-activated sodium and calcium channels), there are at least 77 known genes that encode alpha subunits of potassium channels [1]. Moreover, in contrast to sodium and calcium channels, in which the entire pore-forming channel is formed from one polypeptide, most potassium channels are tetramers that result from the coassembly of either the same or different subunits from the same family. This finding, coupled with the fact that most potassium channel genes have multiple splice isoforms and that the subunits themselves are subject to a variety of posttranslational modifications, means the potassium channel superfamily is capable of producing an extraordinarily large variety of channels with different kinetic behaviors and voltage dependences. 

 The pore-forming *α*-subunits of potassium channel can be divided into voltage-dependent (K_v_) subunits, inward rectifier (K_ir_) subunits, two pore (K_2P_) subunits (which assemble as dimers rather than tetramers), those activated by intracellular calcium (K_Ca_ subunits), and those activated by intracellular sodium (K_Na_ subunits). While the existence of K_Na_ channels has been recognized since the early 1980s, it is only in the last ten years or so that their molecular identity has been known. Research over this time has revealed that rather small changes in the function of these channels can have devastating effects on neuronal development and intellectual function [2, 3]. This review will summarize our current understanding of this class of channels and how they contribute to the electrical and cellular properties of nerve cells. 

## 2. History of K_Na_ Channels

Potassium channels that are activated by an increase in cytoplasmic levels of sodium ions, commonly termed K_Na_ channels, were first reported in cardiomyocytes [7]. Since that time, K_Na_ channels recorded at the single-channel level, or macroscopic currents that can be attributed to K_Na_ channels, have been described in a wide variety of mammalian neurons [8–19], and some of these studies have been reviewed previously [20, 21]. High levels of K_Na_ currents have been found in neurons of the dorsal root ganglion [5, 22, 23]. The existence and general properties of K_Na_ currents have been relatively well conserved throughout evolution and have been extensively studied in larval lamprey spinal cord neurons [24–28]. They have been described in a wide variety of neurons in the nervous system of invertebrates, including antennal-lobe neurons of the sphinx moth, *Manduca sexta* [29], Kenyon cells of mushroom bodies in crickets [30], pressure *P* neurons in the leech [31] and, bag cell neurons that regulate reproductive behavior in the sea hare *Aplysia californica* [32].

 Although the major focus of studies of K_Na_ currents has been in the nervous system, K_Na_ channels have been described in a variety of cardiac cells including guinea pig myocytes and rat myoblasts [7, 33, 34]. They have also been found in guinea pig gastric myocytes [35] mouse diaphragm muscle [36], circular smooth muscle of the opossum lower esophageal sphincter [37], and the thick ascending limb of mouse kidney [38], as well as in Xenopus oocytes [39]. 

## 3. The Slack Channel 

Two genes that encode K_Na_ channels are now known, and these have been termed Slack and Slick, [4, 40, 41]. The term Slack is derived from “sequence like A calcium-activated K channel,” because part of the pore domain and the following S6 domain of Slack is similar to that of the Slo1 (BK) large-conductance calcium-activated potassium channel [40]. Overall, however, there is only 7% identity between Slack and Slo1. The Slack channel has also been termed the Slo2.2 channel and also erroneously listed as K_Ca_4.1. The nomenclature for the human *Slack* gene is KCNT1. 

**Figure 1:**
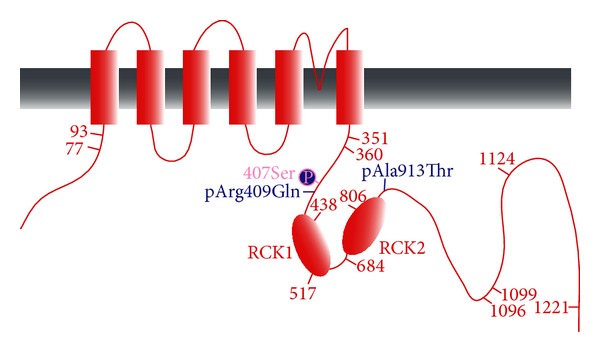
Schematic diagram of a Slack K_Na_ channel subunit depicting the relative positions of the two RCK domains, consensus PKC phosphorylation sites (depicted simply as red numbers corresponding to the positions of the amino acids), the position of the regulatory PKC site Serine 407, and the positions of the two characterized mutations that give rise to malignant migrating partial seizures of infancy (blue) (modified from [2]).

The overall structure of the potassium channel is similar to that of the voltage-dependent K_v_ family of potassium channels in that there are six hydrophobic membrane-spanning domains S1–S6 with a pore P-domain between S5-S6 (Figure 1). With a predicted length of 1237 amino acids, it is, however, very much larger than any of the K_v_ subunits, and the membrane-spanning domains together represent only one seventh of the entire sequence. The majority of the Slack sequence represents a very large cytoplasmic C-terminal domain. Within this C-terminal region there are two predicted RCK (regulators of conductance of K^+^) domains that have also been found in Slo1 channels [42]. X-ray crystallographic studies of the C-terminal domain of chicken Slack channels have confirmed that the structure of the RCK domains is likely to resemble those of Slo1 channels [43]. 

Although the Slack channel was first cloned and expressed in 1998 [40], it was not found to be regulated by internal sodium ions until 2003 [41]. When expressed either in mammalian cell lines or *Xenopus* oocytes and excised into solutions that have equal concentrations of potassium on both sides of the membrane, Slack channels have a relatively large unitary conductance (~180 pS) [4, 41] but the conductance is reduced by over 50% in physiological solutions [40]. As has been found for K_Na_ channels in neurons and other cells, Slack channels in expression systems have multiple subconductance states. 

As is the case for K_v_ and Slo1 subunits, Slack currents increase with depolarization, and, in single-channel recordings, the open probability of Slack channels increases with depolarization. Slack differs from these other channels, however, in that there are no charged residues in the S4 transmembrane domain, which is the principal voltage sensor of these other channels. The residues that confer voltage sensitivity on Slack channels are not yet known. 

**Figure 2:**
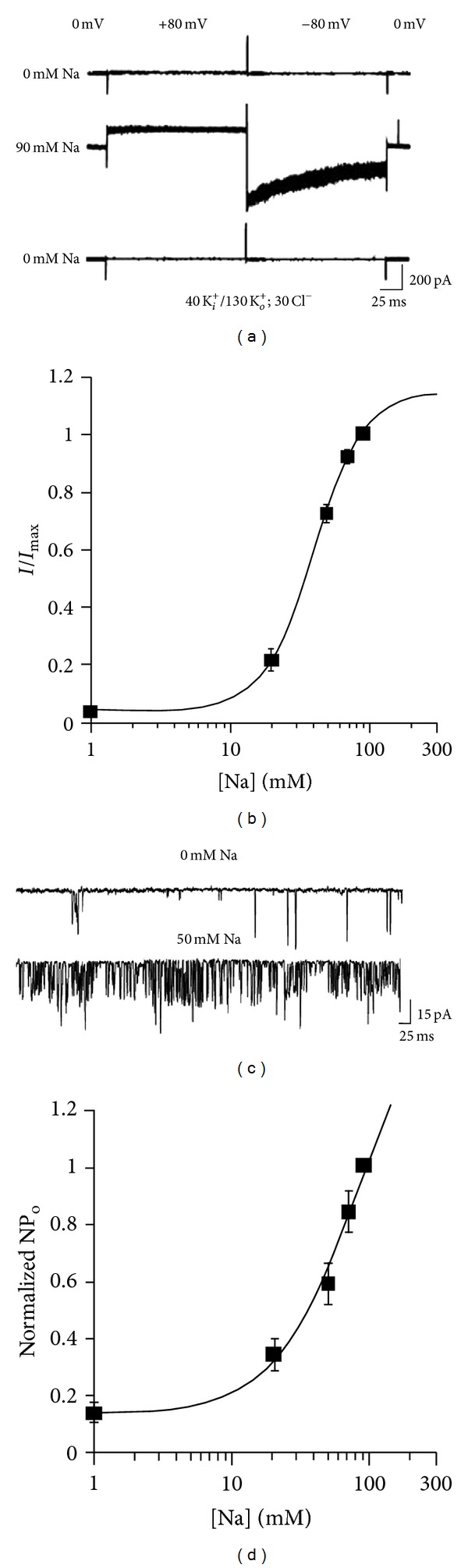
Activation of Slack and Slick channels by cytoplasmic sodium. (a) Representative patch of macroscopic Slack currents recorded at 0 and −80 mV in an excised inside-out patch from Slack-transfected CHO cells in the presence and absence of 90 mM sodium. (b) Concentration-response relationship of sodium activation of Slack current in excised patches. Currents were normalized those recorded in 90 mM sodium. (c) Excised patch recording at −80 mV from a CHO cell transfected with Slick. The cytoplasmic face of the patch was perfused with a solution containing either 0 or 50 mM intracellular sodium. (d) Concentration-response relationship of sodium activation of Slick channels in excised patches. Figures modified from [4].

In excised patches, the half-maximal concentration for activation of Slack channels by sodium ions at the cytoplasmic surface is ~40 mM (Figures 2(a) and 2(b)) [4, 41]. As will be described later, this EC_50_ value may, however, not represent the situation within intact cells [5]. A region within the second RCK domain has been identified as a site that confers sensitivity to sodium ions [44]. This region resembles one found in inwardly rectifying K_ir_3 family channels, which can also be activated by sodium ions [45]. Mutation of either an aspartate residue (D818N) or a histidine (H823A) within this region in Slack lowers sodium sensitivity by an order of magnitude [44]. This finding suggests that, as in Slo1 channels, conformational changes in the RCK domains brought about by ion binding lead to channel opening. 

 Overall the biophysical characteristics of Slack channels in heterologous expression systems resemble those reported for K_Na_ channels in native tissues which have been found to have EC_50_ values for sodium activation of 7–80 mM and to have unitary conductances from 100 to 200 pS, with multiple subconductance states. 

## 4. Slack Isoforms

The rates of activation K_Na_ currents vary substantially in different types of neurons. At least in part, such diversity may arise from different Slack channel subunits that are generated by alternative splicing of *Slack* RNA. Five different transcripts from the rat *Slack *gene have been described, and these are predicted to produce Slack channels that differ in their cytoplasmic amino termini [46]. Two of these transcripts, Slack-A and Slack-B, have been expressed functionally and been found to have very different kinetics of activation. The first isoform to be identified [40], which is now termed Slack-B, has a long N-terminal domain, making it the largest potassium channel subunit currently known. When expressed in *Xenopus* oocytes or mammalian cell lines, Slack-B channels activate slowly over hundreds of milliseconds [4, 40, 41]. 

 The N-terminal domain of the other characterized splice variant, Slack-A, is smaller than that of Slack-B, and the sequence of this N-terminal domain very closely resembles the N-terminus of the other K_Na_ channel subunit Slick (see next section). Neuronal expression of Slack-A and Slack-B channels is driven by independent promoters [46]. In contrast to the very slowly-activating Slack-B channels, Slack-A channels activate very rapidly upon depolarization. The different kinetic behaviors of Slack-A and Slack-B channels are also evident in single-channel recordings in patches excised from *Xenopus* oocytes expressing these subunits. The unitary conductance of the two isoforms is identical. The gating of Slack-A channels is, however, very rapid. During sustained depolarization, brief transient openings to fully open state are interspersed with repeated opening to subconductance states. In contrast, the mean open time of Slack-B channels is about 6 times longer, and subconductance states, although they are very evident, are less frequent than in Slack-A channels [46]. 

The potential effects of the expression of either Slack-A or Slack-B on neuronal firing patterns have been addressed in numerical simulations. Neurons in which potassium currents are dominated by a Slack-A-like current adapt very rapidly to repeated or maintained stimulation. In contrast, Slack-B currents allow neurons to fire rhythmically during maintained stimulation and allow the rate of adaptation rate to vary with the intensity of stimulation [46]. 

## 5. Slick, a Second K_Na_ Channel

The second known gene that encodes a K_Na_ channel was termed *Slick* for sequence like an intermediate conductance K channel, because it is closely related to the *Slack* gene and Slack channels have conductance that is intermediate between that of Slo1 (BK) calcium-activated potassium channels and most other potassium channels [4]. The Slick channel has also been termed the Slo2.1 channel, and the human *Slick* gene is KCNT2. Overall, the sequence of Slick is ~74% identical to that of Slack, and the transmembrane domains and the RCK domains of Slick and Slack are almost identical. The greatest divergence between Slack and Slick occurs at their distal C-terminal regions. 

The predicted cytoplasmic N-terminus of Slick is similar to that of the Slack-A isoform. Consistent with this fact, the characteristics of Slick currents, recorded in mammalian cells or *Xenopus* oocytes, resemble those of Slack-A. Although the unitary conductance of Slick channels (~140 pS) is slightly smaller than that of Slack channels, Slick channels activate very rapidly with depolarization and have multiple subconductance states [4]. The estimated half-maximal concentration for activation of Slick by sodium, 89 mM, is greater than that for Slack channels (Figures 2(c) and 2(d)). Moreover, in contrast to Slack channels, which have an absolute requirement for Na^+^ for channel opening, Slick channels have a basal level of activity in the absence of sodium [4].

 In addition to their sodium sensitivity, both Slick and Slack channels can be activated in increases in intracellular chloride levels [4, 47]. Under physiological conditions, however, Slick channels are markedly more sensitive to this anion [4]. The domains that confer sensitivity of these channels to chloride are not known, and mutations within a region that resembles the “calcium bowl” of Slo1 channels, and which has been established to contribute to the calcium sensitivity of Slo1 channels [48], have not been found to influence the sensitivity of Slack or Slick to either sodium or chloride ions [4]. 

The Slick channel also differs from Slack in having a consensus ATP binding site just C-terminal to the second RCK domain. This site renders Slack channel activity to be sensitive to cytoplasmic ATP levels. The presence of 5 mM ATP reduces Slick whole cell currents, or channel activity in excised patches, [4]. Further description of the effects of ATP on Slick channels will be provided in a later section on physiological modulation of K_Na_ channels.

## 6. Heteromer Formation between Slack-B and Slick Subunits

A variety of lines of evidence indicate that the Slack-B subunit and Slick can coassemble to form heteromeric channels that differ in their properties from those of either subunit expressed alone [49]. Moreover, assembly of heteromeric channels appears to be specific for the Slack-B isoform and does not occur with Slack-A, which, as described above, differs from Slack-B only in its N-terminal cytoplasmic domain. When Slick and Slack-B are coexpressed at a 1 : 1 ratio either in *Xenopus* oocytes or in mammalian HEK293 cells there is an 18–25-fold increase in current amplitude compared to expression of either subunit alone [49]. This increase in current is associated with a comparable increase in levels of the channel subunits in plasma membrane, as assayed by a surface biotinylation assay. No differences in total cellular levels of either protein are, however, found when the subunits are expressed singly or in combination with the other subunit. 

The macroscopic currents of the Slack-B/Slick heteromers differ from those of the homomers [49]. For example, the time for the Slick/Slack-B currents to reach 90% activation is significantly longer than that for either Slick or Slack-B homomeric currents. In addition, the unitary conductance of the Slack-B/Slick channels is intermediate between that of Slack (~180 pS) and Slick (~140 pS). A direct demonstration of this fact was obtained by making a pore mutant of the Slick channel, Slick-EE (Q276E, Y279E), that increases its unitary conductance to ~500 pS. Coexpression of Slick-EE with wild-type Slack-B resulted in channels that had a unitary conductance of ~325 pS, a value that is clearly distinct from that for either channel alone, providing definitive electrophysiological evidence for the formation of Slick/Slack-B heteromers. 

Coimmunoprecipitation experiments have also demonstrated that Slack-B forms a protein complex with Slick in both rat brain tissue and heterologous expression systems [49]. No coimmunoprecipitation could, however, be found for epitope-tagged Slick-A and Slick subunits. This is consistent with electrophysiological findings demonstrating that coexpression of Slack-A with Slick does not increase whole-oocyte currents, compared to expression of either subunit alone, and that no intermediate conductance single-channel activity can be observed when Slack-A is expressed with the Slick-EE mutant. Moreover coexpression of Slack-A with Slick does not increase levels of surface expression of either subunit. 

The specificity of Slack/Slick interactions for the Slack-B isoform is dependent on the extended N-terminal domain of Slack-B. This has been demonstrated by constructing a chimeric channel that replaced the cytoplasmic N-terminal domain of Slick with that of Slack-B. Coexpression of this modified Slick channel with wild-type Slick channels in oocytes produced a 30-fold increase in whole-oocyte currents, which is similar to the increase obtained with Slack-B subunits [49]. Thus the Slack-B N-terminal domain plays a key role in trafficking and/or the level of plasma membrane expression of heteromeric K_Na_ channels.

## 7. Localization of Slack and Slick Channels in the Central Nervous System

 Slack transcripts of about 4.5 kb and 7.5 kb are abundantly expressed in the brain and in the kidney [40]. By the technique of *in situ *hybridization in sections of adult rat brain, Slack is very highly expressed in neurons but no staining is evident in glial cells. Strong hybridization is found throughout the brain, including the cerebral cortex, hippocampus, deep cerebellar nuclei, cerebellar Purkinje cells, reticular tegmental nucleus of the pons, preoptic nucleus, substantia nigra, and auditory brainstem nuclei. Neurons within the thalamus and hypothalamus were found to have a more moderate level of staining [40]. Subsequent quantification of expression of mRNA levels by probes that are specific for either Slack-B or Slack-A isoforms found that both isoforms are present throughout the brain but that the highest levels of both isoforms are detected in brainstem and olfactory bulb [46]. 

Immunocytochemical staining using an antibody to the Slack-B specific N-terminus confirmed the localization of Slack-B in neurons of the brainstem and olfactory bulb of rat [50]. Prominent immunoreactivity was found in the olfactory bulb, red nucleus, and deep cerebellar nuclei, as well as in vestibular and oculomotor nuclei and in the trigeminal system and reticular formation, where intense staining occurs in both cell bodies and axonal fibers. As with the *in situ *hybridization studies, labeling was prominent in auditory brainstem nuclei. Although staining was also evident in neurons of the thalamus, substantia nigra, and amygdala, the only region of the cerebral cortex where the Slack-B isoform was found was in the frontal cortex [50].

 Immunolocalization studies have also been carried out using an antibody targeted against a region in the cytoplasmic C-terminus of Slack [46]. This would be expected to recognize the multiple Slack isoforms that differ at the N-terminus, but not to recognize Slick channels. Immunostaining with this “pan-Slack” antibody found strong labeling of neurons throughout the nervous system of mice. This included the regions previously identified with the Slack-B antibody, as well as other locations such as the dendrites of hippocampal neurons and olfactory bulb glomeruli [46]. 

Messenger RNA for the Slick subunit has a wider distribution throughout the body than does Slack mRNA, and a Slick transcript of ~6.9 kB is expressed in rat heart, where no Slack mRNA has been found [4]. Like Slack however, the highest levels of Slick mRNA are found in the brain. Slick mRNA and immunoreactivity overlap considerably with those for Slack, and are found at high levels in olfactory bulb, midbrain, brain stem, hippocampus, throughout the cerebral cortex with high expression in primary somatosensory and visual regions [51]. Like Slack, high Slick immunoreactivity is found in neurons of sensory pathways including those in the auditory brainstem. The wide colocalization of Slick with the Slack-B isoform suggests that the native K_Na_ channels in these regions are likely to be heteromers of these two subunits. 

Studies have also found Slack channels to be expressed in neurons of the peripheral nervous system. Slack immunolabeling is found in over 90% of neurons of the dorsal root ganglion and has been localized to both the somata and the axonal tracts of small-, medium-, and large-diameter neurons [5, 23]. Localization to axonal tracts has also been detected in electrophysiological experiments on peripheral axons of *Xenopus *neurons, for which a correlation has been found between the localization of K_Na_ channels and voltage-dependent sodium channels [52]. 

## 8. Pharmacology of K_Na_ Channels 

A very common test for the presence of K_Na_ channels in neurons and other cells has been to measure the effects of replacement of external sodium by lithium ions on the net outward current. Lithium is a much weaker activator of K_Na_ channels than sodium. Because lithium readily enters cells though voltage-dependent sodium channels, usually with minimal effects on the inward currents, lithium replacement in voltage-clamp experiments reduces the net outward currents if K_Na_ currents are present. In current clamp experiments, lithium substitution may alter the shape of action potentials or firing patterns in a manner consistent with partial block of K_Na_ currents. The majority of the experiments described later in the section on contributions of K_Na_ channels to neuronal firing patterns have tested the effects of lithium substitution. 

Studies on native K_Na_ channels in cardiac cells found that several antiarrhythmic drugs inhibit these channels in cardiac cells [53–55]. Some of these compounds, such as quinidine, bepridil, and clofilium, have been also found to be very effective and reversible blockers of Slack and Slick channels expressed in oocytes and mammalian cells [4, 56, 57] but are non-specific in that they also act on a variety of other channel types. 

The pharmacological properties of Slack and Slick channels are similar to each other but differ from those of many other potassium channels, including their closest molecular relative, the Slo1 channel. Slack and Slick are only weakly sensitive to the general potassium channel blocker tetraethylammonium ions (TEA) [4]. External barium ions produce a time- and voltage-dependent block of Slack and Slick channels. In contrast, both subunits are insensitive to blockers of calcium-activated potassium channels such as apamin, iberiotoxin, paxilline, and charybdotoxin and to a wide range of blockers of other classes of potassium channels [4, 57]. Although Slick channels are sensitive to cytoplasmic ATP levels, they are insensitive to glibenclamide and diazoxide, an inhibitor and an activator, respectively, of classical K_ATP_ channels [4]. 

A variety of compounds that activate Slack and Slick channels are also known. The first of these to be described was bithionol, which, in Slack-expressing HEK cells, produces a robust increase in the amplitude of Slack currents, with an EC_50_ of 0.77 *μ*M [56]. Similar bithionol-induced increases in current are observed for Slack-expressing oocytes and for native K_Na_ currents in neurons of the auditory brainstem [56, 58]. Bithionol reversibly activates Slack channels even when applied to the extracellular face of excised patches, indicating that it acts on Slack channels relatively directly. The increase in current occurs though a very marked bithionol-induced change in the voltage dependence of Slack channels such that full activation is already observed at potentials as negative as −40 mV [56]. Bithionol is, however, not selective for K_Na_ channels in that it also activates Slo1 calcium-activated potassium channels [56, 59]. 

A screen of pharmacologically active compounds using a rubidium flux assay against Slack channels expressed in CHO cells has revealed other activators [59]. These include riluzole, loxapine, an antipsychotic agent, and niclosamide, an anthelmintic agent. Loxapine was found to be more selective than bithionol in that it has no effect of Slo1 calcium-activated potassium channels. Electrophysiological experiments confirmed that loxapine is effective on recombinant human and rat Slack channels and that it activates native K_Na_ channels in isolated neurons of the rat dorsal root ganglion [59].

## 9. Cellular Regulation of K_Na_ Channels

 The activity of Slack and Slick channels, as well as of heteromeric Slack/Slick channels, is potently regulated by a variety of cellular signaling pathways, including G-protein-coupled receptors linked to activation of PKA or PKC [23, 28, 30, 49, 60, 61], direct phosphorylation of the channels subunits [2, 49, 60], changes in cytoplasmic ATP levels [4], cytoplasmic NAD^+^ levels [5], PIP2 [62], estradiol [63], hypoxia [41, 64], and the fragile X mental retardation protein FMRP [6, 32]. This section will summarize these findings.

### 9.1. Modulation of Slack and Slick by Protein Kinase C

Treatment of mammalian cells or *Xenopus* oocytes expressing Slack channels with diacylglycerol or phorbol ester activators of protein kinase C (PKC) leads to a 2-3-fold increase in current amplitude and a slowing of the rate of activation [2, 60]. This effect of PKC has been found to result from the phosphorylation of a single serine residue S407, located in a PKC consensus phosphorylation site in the “hinge” region of the cytoplasmic C-terminus between the S6 transmembrane domain and the first RCK domain [2]. Mutation of this serine to an alanine (S407A), which renders the site incapable of undergoing phosphorylation, results in apparently normal Slack currents that are entirely insensitive to PKC activation. In contrast, mutation of the twelve other putative PKC phosphorylation sites in the Slack channel (see Figure 1) does not prevent the PKC-induced increase in Slack current [2]. 

 In contrast to Slack channels, the action of PKC on Slick channels is to produce a decrease in current amplitude [60]. As in Slack, this effect is likely to be mediated by a modification within the C-terminus. In particular a chimeric channel that contains the N-terminus and membrane spanning regions of Slack, but the cytoplasmic C-terminus of Slick, responds to PKC activation with a decrease in current. Moreover, direct application of a constitutively active form of PKC directly to the cytoplasmic face of excised patches containing these chimeric channels reduces their activity [60]. Nevertheless the specific sites at which Slick subunits are modified in response to PKC activation are not yet known. 

Heteromeric Slack-B/Slick channels respond to activation of PKC in a manner that is quite distinct from that of either subunit expressed alone [49]. In Xenopus oocytes expressing both subunits, application of PKC activators potently suppresses current by ~90%. This is much greater than the degree of suppression measured for Slick subunits alone (~50%) and cannot therefore be explained by any linear combination of Slick or Slack-B currents. The finding that heteromeric Slack-B/Slick channels are regulated even more potently by PKC activation than are the homomeric channels constitutes one of the many pieces of evidence supporting selective heteromer formation between these subunits [49].

### 9.2. Modulation of Slack and Slick by G Protein Coupled Receptors

 Slack and Slick have been coexpressed in *Xenopus* oocytes with the M1 muscarinic receptor and the mGluR1 metabotropic glutamate receptor [60]. These are G*α*q protein coupled receptors that lead to the activation of PKC and formation of IP3. Accordingly, activation of these receptors leads to an increase in Slack currents and a suppression of Slick currents, consistent with modulation of these channels by PKC. There is widespread colocalization of these receptors with the K_Na_ subunits throughout the nervous system, suggesting that modulation of K_Na_ currents by these receptors is likely to be widespread [60]. Direct modulation of K_Na_ channels by mGluR1 has been observed in native neurons within the lamprey spinal cord [28]. In these cells, activation of mGluR1 produces a strong suppression of current, as expected for Slick or Slack-B/Slick heteromers, and this suppression is prevented by pharmacological inhibition of PKC. Interestingly, chelation of intracellular calcium with the chelator BAPTA (1,2-bis(2-aminophenoxy)ethane-*N,N,N*′,*N*′-tetraacetic acid) prevented modulation of the K_Na_ current by PKC but unmasked an mGluR1-dependent activation of K_Na_ current by an independent pathway whose mechanism is not yet known [28]. 

 Modulation of Slack channels by G protein-coupled receptors has been studied using a novel label-free technology to detect changes in the distribution of mass close to the plasma membrane [61]. The characteristic change in mass that normally follows activation of native receptors in HEK-293 cells is significantly modified in amplitude and timing by the coexpression of Slack channels, further supporting the finding of modulation of K_Na_ current by these receptors [61].

### 9.3. Modulation of K_Na_ Currents by Cyclic AMP

The activity of K_Na_ channels in Kenyon cells isolated from the mushroom body of the cricket (*Gryllus bimaculatus*) is enhanced upon application of either the neurotransmitter octopamine or the membrane-permeable analog cyclic AMP analog 8-Br-cyclic-AMP [30]. The increase in channel activity was found to be attenuated by the protein kinase A (PKA) inhibitor H-89. Conversely, K_Na_ channel activity in these cells is decreased by the transmitter dopamine or by the cyclic GMP analog 8-Br-cyclic GMP, and this decrease in open probability is antagonized by the protein kinase G (PKG) inhibitor KT5823. These findings have led to the proposal that modulation of K_Na_ currents play a role in olfactory learning circuits that mediate reward and punishment signals in insects, which are regulated by octopamine and dopamine, respectively [30]. 

The Slack subunit is expressed at high levels in primary afferent nociceptor neurons located in the dorsal root ganglion [5, 23] and K_Na_ currents can readily be recorded in these cells [5, 22, 23]. Inflammatory substances such as prostaglandin E2 that activate the PKA pathway greatly increase the excitability of these neurons, sensitizing them to thermal and mechanical stimulation. In their resting state, the neurons adapt rapidly to maintained depolarizations, typically generating only one or two action potentials at the onset of depolarization. After an elevation of cyclic AMP levels that activates PKA, adaptation is greatly reduced such that sustained firing can be recorded throughout a prolonged depolarization. In major part, the effects of PKA can be attributed to a decrease in Slack K_Na_ current, and suppression of Slack expression using anti-Slack siRNA produces a reduction in adaptation similar to that produced by PKA activation [23].

In contrast to the effects of PKC on Slack channels that were described above, the effects of PKA on Slack are indirect and do not involve phosphorylation of the channel subunit itself [23, 65]. Slack channels in HEK-293 cells are insensitive to cyclic AMP analogs or to the PKA inhibitor KT5720, and application of the catalytic subunit of PKA to the cytoplasmic face of Slack channels excised from transfected cells or dorsal root ganglion neurons has no effect on channel opening. Instead, activation of PKA decreases Slack current amplitude by rapidly internalizing Slack channels from the plasma membrane into internal organelles [23]. Such internalization was detected by confocal microscopy of neurons expressing Slack channels tagged at their N-terminus with the green fluorescent protein GFP. Elevations of cyclic AMP reduced colocalization of the labeled channels with a cell surface marker. A reduction in native Slack channels at the plasma membrane following PKA activation was also confirmed using a surface biotinylation assay [23]. 

### 9.4. Modulation of Slack and Slick by PIP2

A variety of ion channels have been shown to be regulated by phosphatidylinositol 4,5-biphosphate (PIP2), a phospholipid that is, localized to the inner lipid leaflet of the plasma membrane [66]. Exogenously applied PI(4,5)P2, as well as its isoform PI(3,4)P2, increases the amplitude of Slack and Slick currents expressed in *Xenopus* oocytes, and pharmacological agents that are expected to reduce endogenous PIP2 levels reduce current amplitude [62]. Regulation by PIP2 was found to require specific lysine residues in the C-termini of the channels. Mutation of these lysines to alanine (K339A in Slack and K306A in Slick) produced functional K_Na_ currents that were insensitive to the activating effects of PIP2 [62]. 

### 9.5. Suppression of Slick Channels by Cytoplasmic ATP

The Slick subunit differs from Slack in that it contains a consensus ATP binding site just C-terminal to the second RCK domain [4]. The current density of Slick currents, but not Slack currents, is reduced by ~80% by the presence of 5 mM ATP at the cytoplasmic face of the channels. The same full effect is observed with ATP*γ*S, a slowly hydrolyzable ATP analog, and the nonhydrolyzable analog AMP-PNP but not with ADP. Suppression by ATP of Slick currents at all membrane voltages can also be readily detected in excised patches [4]. 

 Confirmation that the effect of ATP on Slick channels is mediated by the consensus ATP binding site in the C-terminal domain was obtained by site-directed mutagenesis to replace the glycine at residue 1032 in this site with a serine [4]. The mutant G1032S Slick channel was fully functional but failed to decrease channel activity in response to ATP. The sensitivity of Slick channels to ATP levels suggests that their activity could be enhanced during periods of high metabolic demand when cellular ATP levels fall.

### 9.6. Modulation of Slack by NAD^+^

 K_Na_ channels can readily be detected in cell-attached patch-clamp recordings on neurons [5, 16, 58]. This may be surprising given that the cytoplasmic sodium concentration in resting cells is relatively low (<~10 mM, see Section 10 below) and, as described earlier, the EC_50_ for activation of these channels in excised patches is relatively high (typically ~40–80 mM). This discrepancy is resolved by the finding that the open probability and sodium sensitivity of K_Na_ channels are greatly reduced following excision of membrane patches [5, 16], suggesting that rundown of K_Na_ activity in excised patches may result from loss of some cytoplasmic factor. 

**Figure 3:**
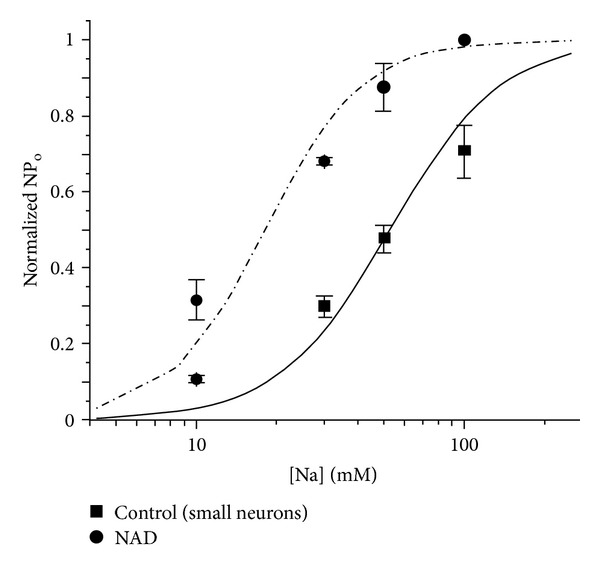
NAD^+^ increases the sodium sensitivity of K_Na_ channels excised from adult rat dorsal root ganglion neurons. Concentration-response relationship provides estimates of EC_50_ for sodium activation of 50 mM and 17 mM in the absence and presence of NAD^+^, respectively. Figure modified from [5].

 A major cytoplasmic factor that regulates both Slack and Slick channels has been found to be nicotinamide adenine dinucleotide NAD^+^ [5]. Both subunits have a putative NAD^+^-binding site within their second RCK domain. Application of NAD^+^ to the cytoplasmic face of K_Na_ channels excised from dorsal root ganglion neurons increases their open probability and reduces the measured EC_50_ for activation of sodium from ~50 mM to ~17 mM (Figure 3). NAD^+^, as well as NADP^+^ but not NADH, also increased the open probability of Slack channels excised from a transfected mammalian cell line. Mutation of glycine in the putative NAD^+^-binding site of Slack (G792A) resulted in a loss of the ability of NAD^+^ to potentiate activity [5]. These findings suggest that K_Na_ channels play a much more prominent role in the normal physiology of excitable cells than would be suggested by their properties in isolated patches.

### 9.7. Modulation of Slack by Estradiol

 The open probability of channels reconstituted from lipid bilayers from extracts of rat cortex has been found to be increased by 17*β*-estradiol [63]. Although these channels were not explicitly shown to be K_Na_ channels, a similar effect was reported for whole-cell currents recorded in Slack-expressing HEK-293 cells. Activation was not prevented by tamoxifen, a blocker of classical estrogen receptors, but may be a consequence of direct binding of 17*β*-estradiol to Slack channels themselves [63]. 

### 9.8. Modulation of Slack Channels by Hypoxia

 During hypoxic conditions, cytoplasmic levels of internal sodium and chloride ions, as well as those of H^+^ and CO_2_, rise. It has been suggested that activation of K_Na_ channels could act to hyperpolarize cells during hypoxia, providing a protective mechanism [7]. This conjecture is supported by the finding that nematodes lacking the *Slack* ortholog in this species are significantly more sensitive to hypoxic death than are wild-type animals [41]. Further support for a role for Slack channels in the response to hypoxia comes from recordings of Slack channel activity in *Xenopus* oocytes exposed to increasing concentrations of H^+^ and CO_2_, demonstrating that elevations of sodium activate Slack channels under cellular conditions comparable to those expected in ischemia/hypoxia [64].

### 9.9. Interactions of Slack with the Fragile X Mental Retardation Protein FMRP

 Fragile X syndrome, the most common inherited form of intellectual disability in humans, is caused by loss of expression of the RNA-binding protein FMRP (fragile X mental retardation protein). FMPR is known to be required for normal activity-dependent protein translation in neurons. Yeast two-hybrid and coimmunoprecipitation experiments have shown that the cytoplasmic C-terminus of the Slack subunit interacts with this protein [6]. Moreover Slack-FMRP complexes also contain messenger RNAs that are targets of FMRP, such as the mRNAs encoding Map1b and Arc. No association of mRNAs with Slack can, however, be found in mice lacking FMRP, indicating that these are bound to Slack despite its interaction with FMRP. A peptide array assay revealed confirmed that FMRP binds selectively to sequences at the distal C-terminus of Slack [6].

**Figure 4:**
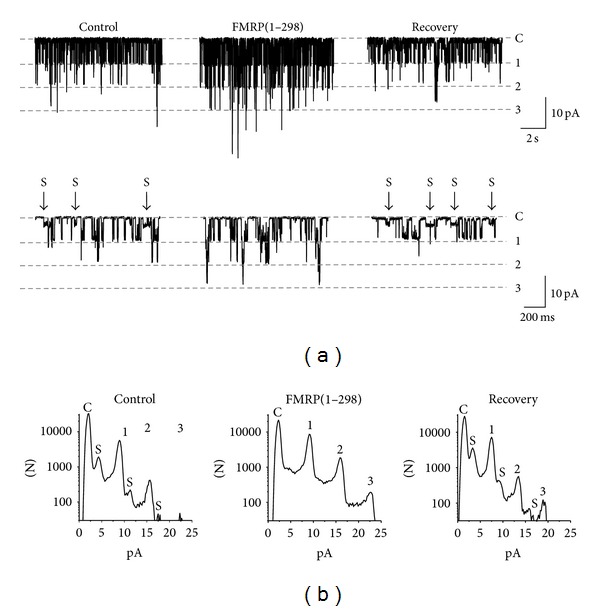
FMRP alters gating of Slack-B channels in inside-out patches. (a) Recording of a patch containing 3-4 Slack channels before, during and after transient application of FMRP(1–298) to the cytoplasmic face of the patch. Top traces show representative 10 s examples of recording at −40 mV and bottom traces show expanded time views of the same patch. Arrows marked S indicate the occurrence of subconductance states that are suppressed by FMRP(1–298). (b) All-points amplitude histograms of Slack channel activity plotted on a logarithmic scale before and after application of FMRP(1–298) and after washout. C marks the closed state, S indicates subconductance states, and numbers on peaks in the histograms refer to the number of fully open Slack channels. Modified from [6].

 FMRP is a potent activator of Slack channel activity [6]. Application of recombinant FMRP(1–298), which contains the majority of protein-protein interaction domains of FMRP, to the cytoplasmic face of Slack channels in excised patches reversibly increases channel opening by two- to threefold (Figure 4(a)). This is associated with the almost complete elimination of openings to subconductance states, which are prominent in untreated patches (Figure 4(b)). FMRP was found to have no effect on the channel activity of a Slack truncation mutant (Slack-BΔ804), which lacks sites found to interact with FMRP in biochemical assays [6]. 

 Support for a role for FMRP in regulating the amplitude of native K_Na_ currents has also come from recordings of neurons from animals lacking the *fmr1* gene, which encodes FMPR [6]. As will be described later, K_Na_ channels account for a major component of potassium current in principal neurons of the medial nucleus of the trapezoid body (MNTB). While loss of FMRP does not affect total levels of Slack protein, the level of the K_Na_ component of current in these cells is substantially reduced in the FMRP knockout mice compared to that in wild-type animals, consistent with an activating role for this RNA-binding protein [6].

The interaction between Slack and FMRP is likely to be evolutionarily conserved. Both proteins have been found to be coexpressed in bag cell (BC) neurons, which regulate reproductive behaviors in mollusk *Aplysia* [32]. FMRP and Slack immunoreactivity are colocalized at the periphery of isolated BC neurons and the two proteins can be reciprocally coimmunoprecipitated. The native Slack current in these cells was identified using an siRNA approach, and intracellular injection of FMRP(1–298) induced a K_Na_ current that in its properties matches the Slack current. As for Slack channels in heterologous expression systems, addition of FMRP(1–298) to the inside-out patches containing native *Aplysia* K_Na_ channels increased channel opening. In current clamp recordings, FMRP(1–298) produced a narrowing of action potentials.

 Downregulation of Slack expression in BC neurons using an siRNA approach had an interesting effect on long-term changes in the excitability of these cells [32]. In untreated cells, brief stimulation of these neurons produces a characteristic prolonged (~30 min) discharge that is followed by a prolonged (~18 hr) inhibitory state. Recovery from this inhibitory state requires new protein synthesis and is blocked by the protein synthesis inhibitor anisomycin. Anti-Slack siRNA did not alter the ability of BC neurons to undergo the normal discharge but, like inhibition of protein synthesis, prevented recovery from the inhibitory state [32]. Although other interpretations are possible, these findings raise the possibility that the activity of Slack channels is linked to the regulation of protein translation through the FMRP-RNA pathway. 

 As will be described later, point mutations in human Slack channels can produce devastating effects on intellectual development [2, 3]. A variety of experiments have demonstrated that activation of ion channels can influence cytoplasmic processes independently of ion flux through the channels [67]. The interaction between the C-terminus of Slack and FMRP raises the hypothesis that conformational changes in the C-terminus could represent such a “nonconducting” function of Slack that links neuronal firing to changes in protein translation.

## 10. Sodium Entry Pathways That Activate K_Na_ Channels 

 Estimates of cytoplasmic sodium concentrations in resting unstimulated neurons, obtained using sodium-sensitive microelectrodes and ratiometric dyes, provide values between ~4 mM and 15 mM [68]. As noted earlier in the section on NAD^+^ regulation of Slack channels, this may be sufficient to provide significant activation of native K_Na_ channels in intact neurons even under resting conditions [5]. Internal sodium levels rise in response to stimulation, principally as a result of entry through voltage-dependent sodium channels and ionotropic ligand-gated receptors such as AMPA and NMDA glutamate receptors [69]. Stimulation, particularly repetitive tetanic stimulation, produces local increases in sodium concentrations that can rise to levels between 45 and 100 mM [70, 71]. In particular high levels can be attained in restricted compartments such as active dendritic spines.

 Although the upstroke of action potentials in most neurons is driven by rapidly-inactivating voltage-dependent sodium currents, these currents are transient. Evidence suggest that the much smaller persistent component of sodium current that does not inactivate with maintained depolarization is responsible both for sustained elevations of internal sodium during stimulation [69] and for selective activation of K_Na_ channels [72, 73]. It has been found that, in patches excised from somata of rat neurons, sodium entry through persistent tetrodotoxin-sensitive sodium channels is the dominant factor that leads to activation of K_Na_ channels in the same patch [73]. Activation of K_Na_ channels by sodium entry in the isolated patches was enhanced by veratridine, which prolongs sodium channel openings, and was observed in the absence of sodium at the cytoplasmic face of the patches. Moreover, activation appeared to be independent of the amplitude of the much larger transient sodium current [73]. Although the molecular basis for persistent sodium channels is not fully understood, further support for a selective activation of K_Na_ currents by entry through persistent channels has also been found in electrocytes of electric fish, in which the time course of K_Na_ currents during depolarizations reflects that of the persistent sodium current, and not that of the very much larger transient sodium current [74].

 A number of studies have documented that K_Na_ currents are also activated by sodium entry through ionotropic glutamate receptors, particularly AMPA receptors. Slack subunits can be coimmunoprecipitated with GluR2/3 AMPA receptors using rat brain synaptosomal fractions or lysates of the lamprey central nervous system [27]. Moreover, Slack subunits have been found to bind directly to the PDZ domain of PSD-95 (postsynaptic density-95 protein), a major component of the postsynaptic density of glutamatergic synapses, and to colocalize with PSD-95 in cultures of mouse cortical neurons [75]. In electrophysiological studies of lamprey neurons, application of AMPA was been found to activate a K_Na_ current with pharmacological properties of Slack [25]. Activation of this K_Na_ current regulates both the amplitude and the decay rate of AMPA-induced synaptic currents providing a feedback loop that decreases the size of glutamatergic postsynaptic potentials [27].

 Activation of K_Na_ currents following activation of AMPA receptors has also been proposed in studies of neurons of the locus coeruleus, to which application of glutamate produces a burst of action potentials followed by period of inhibition lasting 30–45 seconds [76]. The potassium current responsible for the period of inhibition was selectively activated by AMPA or the AMPA-receptor agonist quisqualate, but not by other stimuli that excite these neurons. Consistent with this current being a K_Na_ current, it was attenuated by lowering of external sodium concentrations but not affected by external calcium levels, and it was potentiated by the K_Na_ channel opener bithionol [76].

While persistent voltage-dependent sodium channels and AMPA receptors may represent the primary sodium entry pathways for activation of K_Na_ channels, there exist other pathways that allow sodium ions to enter excitable cells and whose role has been less explored. Slack subunits are abundantly expressed in mitral cells of the olfactory bulb [46, 50]. In these neurons, deletion of the gene for a major voltage-dependent potassium channel Kv1.3, produces a compensatory increase in levels of Slack-B protein and in Slack K_Na_ currents, identified using an anti-Slack siRNA technique [77]. Activation of the K_Na_ current during depolarization of these cells could only be significantly suppressed by a combination of tetrodotoxin, to block voltage-dependent sodium channels, and ZD7288, a blocker of H-channels, which are nonselective cation channels that flux both sodium and potassium ions. Either drug alone failed to significantly suppress K_Na_ currents, suggesting that sodium entry through H-channels plays a role in K_Na_ channel activation in these neurons [77]. 

## 11. Contribution of K_Na_ Channels to Neuronal Firing Patterns

The component of whole-cell potassium current in neurons that can be attributed to Slack channel subunits has been identified in mitral cells of the olfactory bulb and medium spiny neuron of the striatum using siRNAs to suppress translation of Slack mRNA [72, 77]. Such experiments indicate that Slack K_Na_ channels are responsible for a very major component of the total delayed outward current in neurons under physiological conditions [72]. The specific contributions that K_Na_ channels make to the electrical personality of individual types of neurons may differ in different cell types.

 One of the earliest effects to be described for activation of K_Na_ currents is to produce a slow afterhyperpolarization (sAHP) following a burst of action potentials. This was first described in pyramidal neurons of the cat sensorimotor cortex [11, 78], and similar slow sodium-dependent sAHPs that endure for many seconds have been found in a wide variety of neuronal types [79–84]. These have been the subject of earlier reviews [20, 21]. Such slow potentials have been found to influence the timing of regular bursts of action potentials, such as the 3–12 per minute spindle waves that occur at the onset of sleep in certain neurons within the thalamus [80]. Slow activation of K_Na_ during repetitive firing also produces an adaptation of firing rate during bursts of action potentials [23] and such adaptation is predicted by numerical simulations that incorporate K_Na_ currents [46, 85]. 

 In some neurons, such as neocortical pyramidal neurons and hippocampal CA1 neurons, a significant amount of K_Na_ current can also be activated by single spikes and contribute to action potentials repolarization, or to the amplitude of depolarizing afterpotentials that follow single spikes and are generated by persistent sodium currents [83, 86]. In these cases, K_Na_ current activation may be detected within ~5 msec following a single-action potential. A recent study has examined the mechanism by which the cells of the electric organ (electrocytes) of certain electric fish are able to generate sustained firing at frequencies greater than 500 Hz [74]. Slack channels are expressed at high levels in these cells and their delayed potassium current is almost exclusively a K_Na_ current that is activated by a persistent sodium current. Numerical simulations have indicated that the use of K_Na_ current to repolarize the very large action potentials of these cells allows the cells to fire at such high rates with minimal energy expenditure [74].

 A set of neurons that have been particularly instructive in studies of the role of K_Na_ channels in shaping neuronal firing are those that control locomotion in the spinal cord of lamprey [24–28, 87]. In addition to the transient K_Na_ current that is activated by AMPA receptors and that was described earlier [25, 27], these neurons appear to have two distinct components of K_Na_ current that are triggered by action potential firing—a transient component that follows a single spike and a slower more sustained component [24, 26, 87]. The rapid transient K_Na_ current is activated by the sodium influx through tetrodotoxin-sensitive channel during the action potentials, and may play a role in determining the amplitude and duration of individual action potentials. The sustained K_Na_ current is not abolished by tetrodotoxin, suggesting that it is selectively activated by an independent sodium entry pathway and may underlie the calcium-independent sAHP that occurs during repetitive bursting in lamprey spinal cord neurons [24]. 

Experiments with neurons that fire at high rates with very high temporal accuracy have provided another potential biological role for K_Na_ channels [58]. Slack and Slick are expressed at high levels in neurons of the medial nucleus of the trapezoid (MNTB) within the auditory brainstem [6, 50, 51]. These neurons fire at rates up to ~800 Hz, and accurate timing of their action potentials is required for transmission of information about the localization of sound stimuli. K_Na_ channels are readily be detected in both cell-attached and excised patches on the somata of MNTB neurons and they are localized appropriately for activation by sodium influx through AMPA receptors or voltage-dependent sodium channels [58]. Current-clamp recordings from MNTB neurons and numerical simulations indicate that, as K_Na_ channels become activated, temporal accuracy of firing is improved. This occurs because the voltage-dependence of Slack and Slick channels allows them to open near the resting potential of MNTB neurons, increasing the resting conductance and decreasing the membrane time constant. An increase in temporal fidelity of MNTB neurons can also be produced by treatment with the pharmacological K_Na_ activator bithionol [58]. 

## 12. Mutations in Slack Channels Result in Epilepsy and Intellectual Disabilities

*De novo* human mutations that produce single-amino-acid alterations in Slack channels produce devastating effects on development and intellectual function [2, 3]. One set of Slack mutations results in malignant migrating partial seizures of infancy (MMPSI) [2]. This disease is characterized by pharmacoresistant seizures in the first 6 months of life that arise randomly and “migrate” from one brain region to another. While the occurrence of seizures abates with age, MMPSI results in near-total arrest of psychomotor development. Exome sequencing revealed that at least 50% of MMPSI patients have mutations in the large cytoplasmic C-terminal domain of Slack [2]. 

Two mutations in the human Slack gene that produce MMPSI are R428Q and A934T, which correspond to the mutations R409Q and A913T in the highly conserved rat Slack sequence [2]. Expression of these mutants in *Xenopus* oocytes produced Slack currents with voltage dependence and kinetic behavior that matched that of wild-type Slack channels but with a current amplitude that was increased 2-3-fold over that of wild-type Slack. This increase in current likely results because the mutations produce channels that are either more prone to undergo phosphorylation by PKC (or less likely to be dephosphorylated) or that mimic the conformation induced by PKC activation. One of the MMPSI mutations R409Q lies adjacent to the phosphorylation site S407 that, as described earlier, enhances current by 2-3-fold when phosphorylated by PKC. Consistent with this interpretation, current amplitude of the two MMPSI mutations could not be further increased upon PKC activation [2]. In other respects the MMPSI currents resembled that of wild-type channels, with unitary conductance and sodium dependences matching those of wild-type Slack. Both MMPSI mutant channels, however, had reduced openings to subconductance states compared to the wild-type channels [2].

A second disease that has been attributed to a different set of mutations in the C-terminal domain of Slack channels is autosomal dominant nocturnal frontal lobe epilepsy (ADNFLE) [3]. This is a childhood-onset focal epilepsy syndrome in which motor seizures arise during sleep. Onset of ADNFLE in the patients with Slack mutations occurred at a mean age of 6 years and was associated with mild to severe intellectual disability and/or severe psychiatric problems. The effects of these ADNFLE mutations on the function of Slack channels have, however, not been investigated. Interestingly, another known genetic cause of ADNFLE is mutations in subunits of the neuronal nicotinic acetycholine receptor. The acetycholine receptor ADNFLE mutations do not, however, produce intellectual disability, which contrasts strikingly to the effects of the Slack mutations [3]. 

It is possible that the very severe outcomes of mutations in Slack occur solely because of changes in the amplitude of Slack currents. An increase in total potassium current in rapidly firing interneurons could reduce the firing rate of these cells, predisposing the nervous system to hyperexcitability. The relatively modest increase in current that occurs with the MMPSI mutations [2], however, suggests that other processes may also be affected. As described earlier, the large C-terminal cytoplasmic domain of Slack, in which MMPSI and ADNFLE mutations are located, interacts with FMRP and its cargo mRNAs [6]. Thus it is possible that, in addition to directly altering the excitability of central neurons, mutations in Slack disrupt the regulation of activity-dependent protein translation in neurons. 

## 13. Conclusions

This review has covered the molecular and cellular properties of Slack and Slick, two potassium channel subunits that provide neurons and other cells with K_Na_ channels. It should be pointed out that additional potassium channels that are activated by cytoplasmic sodium may also exist. For example, some of the native K_Na_ currents that have been described in neurons do not match Slack or Slick in their single-channel conductances or pharmacological properties [88]. These may perhaps represent inward rectifier potassium channels that are activated by sodium [45, 89], and the physiological roles of the sodium sensitivity of these channels require further investigation. Nevertheless, Slack and Slick, which are very widely expressed in the brain, appear to play dominant roles in shaping the firing properties of very many types of neurons. Human mutations in these channels produce profound effects on neuronal function and development, suggesting that perhaps their biological role may extend beyond simply setting the level of neuronal excitability, and that these channels may influence cytoplasmic biochemical pathways that regulate development, plasticity, and intellectual function.
